# Measuring Cognition Load Using Eye-Tracking Parameters Based on Algorithm Description Tools

**DOI:** 10.3390/s22030912

**Published:** 2022-01-25

**Authors:** Jozsef Katona

**Affiliations:** CogInfoCom Based LearnAbility Research Team, Department of Software Development and Application, Institute of Computer Engineering, University of Dunaujvaros, 2400 Dunaujvaros, Hungary; katonaj@uniduna.hu; Tel.: +36-25-551-605

**Keywords:** cognitive task, cognition load, eye-tracking, programming, algorithm description tools

## Abstract

Writing a computer program is a complex cognitive task, especially for a new person in the field. In this research an eye-tracking system was developed and applied, which allows the observation of eye movement parameters during programming as a complex, cognitive process, and the conclusions can be drawn from the results. The aim of the paper is to examine whether the flowchart or Nassi–Shneiderman diagram is a more efficient algorithm descripting tool for describing cognitive load by recording and evaluating eye movement parameters. The results show that the case of the interpreting flowchart has significantly longer fixation duration, more number of fixations, and larger pupil diameter than the case of the Nassi–Shneiderman diagram interpreting. Based on the results of the study, it is clear how important it is to choose the right programming tools for efficient and lower cost application development.

## 1. Introduction

Writing a computer program is a complex cognitive task, especially for a new person in the field [1]. In order to facilitate the process and to understand the algorithms to be implemented a higher level of algorithmic thinking and problem solving is required [2,3,4]. Algorithm description tools have been introduced to describe the algorithm to be implemented independently of the programming language in order to make the algorithms more transparent and understandable. The commonly used algorithm description tool is the flowchart (FCh), which describes the algorithm as a directed graph, thus illustrating the steps of execution, while the Nassi–Shneiderman diagram (NSD), also called NS diagram or structogram, represents the algorithm as a graph without edges as a “structured flow charts”. Additional algorithm description tools have recently been applied like the pseudocode, Jackson diagram, the sentence-like description and the description with tree; however, the current study performs the analysis of the previously mentioned FCh and NS diagrams.

In the visual programming described in the article by Charntaweekhun and Wangsiripitak [1], students learning programming can simply compile and run a program using the FCh without any coding and allow for easy debugging and detection. Thus, the presented system is excellent for teaching structural programming, as it avoids the learning difficulty caused by the individual syntax of programming languages, thus providing an opportunity to develop problem-solving skills [1,5].

In the study, Xinogalos [5] provides an overview of FCh-based programming environments and makes suggestions for making software engineering education more effective. In the article in Cabo [6], it was found that students who use FCh to solve problems effectively will learn Python or similar programming languages more easily (r-squared = 0.68). Therefore, the use of flowcharts in programming education could appear as a kind of supportive tool in the development of various cognitive processes [6]. Hooshyar et al. [7] aimed to provide beginner programmers with a framework in which to develop their problem-solving skills, as they found that the weakness of this skill was directly related to the syntax of the programming language to be learned and the development environment used. However, the use of FCh can not only mean more efficient knowledge transfer in the field of education but can also be seen as a supportive tool in industry and in different areas of research.

Shafeek and Karunarathne [8] explain that in a modern distributed software development environment, communication between developers is mostly the source code, which is thus difficult in many cases. In the research, the authors implemented a software prototype that can turn source code into an FCh, thus overcoming communication difficulties between developers, as source code can be read and interpreted in the form of independent, easy-to-understand diagrams [8].

In the research, Ying and Feng [9] describe a low-level FCh-based language that can be used to implement high-level quantum languages and design quantum compilers. The formal semantics of the FCh language was formulated and the notion of the correctness of programs written in that language was introduced.

**Figure 1:**
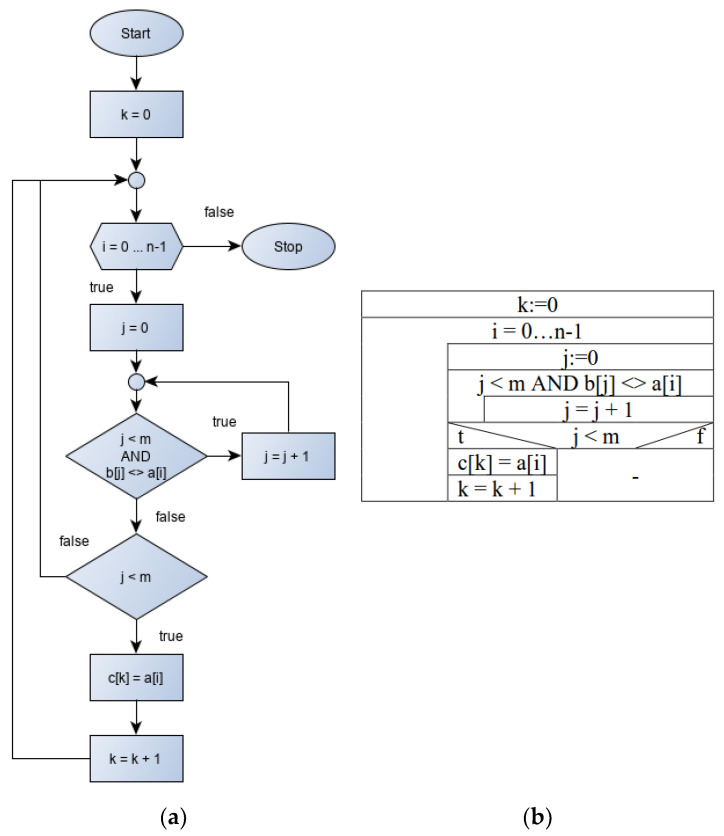
The FCh (**a**) and the N–S or the structogram diagram (**b**) of Intersection algorithm.

The literature reviewed above also shows that the use of FChs is of principal importance in the development of cognitive processes, communication and other areas of research as well. However, the disadvantage of FChs is that they can easily become large and difficult to fix. These disadvantages can be overcome with the NS diagram, as the entire algorithm can be represented without edges. Figure 1 shows the same algorithm description with the FCh and the NS diagram. The difference in size is clearly visible.

However, the difference in size does not necessarily mean that the NS diagram is easier to understand. If the intelligibility and readability of the tools describing each algorithm could be measured in an accurate and objective way, it could be of help in selecting the appropriate tool, which would be of great support, especially for beginning programmers, in learning more complex algorithms to create systems that define the technology of the society of the future [10].

Summarizing Charntaweekhun and Wangsiripitak [1], who used the FCh for syntax-independent visual programming, Xinogalos [5] made suggestions for using the FCh for more effective software development education; Cabo [6] and Hooshyar et al. [7] see the FCh as a tool to support the development of cognitive processes; for Shafeek and Karunarathne [8] communication is easier to implement between developers than the source code based sort; Ying and Feng [9] said the FCh can help with high-level quantum programming. However, I examined the intelligibility of the FCh using eye movement parameters and compared it with the intelligibility of the N-S as since these algorithm description tools have not been examined by anyone else in this way. All in all, the article examines that the NS diagram can be read and interpreted with less or more cognitive load, and the implementation of the algorithms can be done with a lower or higher mental load.

## 2. Theoretical Background

There are basically three main approaches to measuring cognitive load [11]. The first method is to analyze performance indicators, such as the total score achieved in a task [12,13], or the time spent on a task [14]. However, the disadvantages of this type of measurement are that performance indicators need to be personalized to the task and monitoring of continuous cognitive force is not feasible either, as scores are obtained at certain intervals or after completion of a task. The second method is the questionnaire approach, which provides information about the difficulty of the task on the basis of subjective measurement; moreover, in this case we get these opinions mostly after the completion of the task [15]. The most reliable technique is based on physiological observation, which allows us to obtain real-time information about a person’s cognitive load without having to interrupt the task-solving process [16].

Non-invasive psychophysical tools provide an opportunity for researchers to measure a person’s emotional states [17], attention [18] or cognitive load in real time. Recently, affordable and high-resolution eye movement tracking devices have been developed to record eye movement parameters and help the eye-tracking-based research [19]. These devices produce the main eye-tracking parameters such as fixations (relatively stable focus of eyes on the element of the presented object), saccades (quick movements between 30 and 120 milliseconds of the eyes between two fixation points) and pupil diameter [20]. These parameters can provide information about a test subject’s behavior, level of visual attention or cognitive status [21]. Conclusions can be drawn from, for example, examining how long a given person looks at a given object on the screen (fixation duration) and how many times (number of fixations) [22]. If someone spends a lot of time in a given position (the duration of the fixation is long), it may indicate that they have difficulty interpreting the information in a given position [20,21,23,24]. In addition, knowing the duration of fixation, we can also create a so-called attention map, which allows further analyses [25]. The results of Just and Luftos [26,27,28] show that a relationship can be discovered between eye fixations and cognitive load, according to which the more and longer the number and duration of fixations, the greater the cognitive load. In addition to the examination of fixations, pupillometry is widely used to examine cognitive load, since under constant light conditions, the size of the pupil changes systematically to the limit of mental effort [29,30,31]. For example, at focusing and concentration under constant light conditions, the diameter of the pupil gradually increases [32].

The Area of Interest (AOI) is a distinguished, high-priority area that can be examined separately. Indicators used in AOI analyses, such as the length and number of fixations and the number of visits (returns to the area), were the most commonly used, as they may also indicate the subjective importance of a particular area [20,21,22,23]. Furthermore, the higher the number of visits to the AOI area, the higher the cognitive load on processing the information there [20,21,22,23,33].

All in all, the following research questions are addressed in this study:

Research Question 1 (RQ1): Is there a significant difference in the visual parameters (fixation duration mean, number of fixations and average of pupil diameter) in the interpretation of the FCh and the N-S?

Research Question 2 (RQ2): How are visual parameters (fixation duration mean, number of fixations and average of pupil diameter) distributed over implementation when test subjects are using FCh compared to N-S?

## 3. Materials and Methods

In the research, the eye movement parameters of test subjects were recorded using a GazePoint 3 (https://www.gazept.com/product/gazepoint-gp3-eye-tracker/ (accessed on 27 December 2021)) eye-tracker tool and the OGAMA (http://www.ogama.net/ (accessed on 27 December 2021)) open source software package while implementing a randomly selected algorithm on an FCh or N-S diagram in a C# programming language in a Visual Studio development environment.

### 3.1. Test Subjects

The study involved 42 test subjects (12 woman and 30 man) between the ages of 18 and 22 (*M* = 19.8, *SD* = 1.74) who were not on medication and declared themselves completely healthy, with no psychiatric or neurological disorders and with no difficulties in reading or learning in the past and during the test and who applied for the test on a voluntary basis. Based on the results of the programming subject, those with similarly good, better-than-average programming skills were selected and knew the tools used in the study.

### 3.2. Test Conditions and Steps of the Research

**Figure 2:**
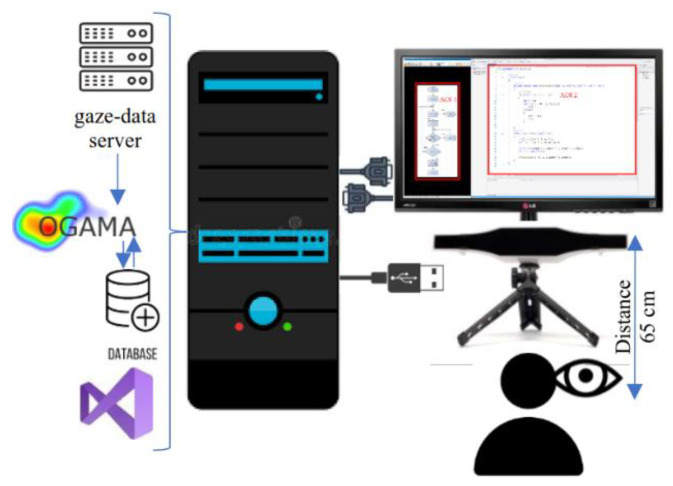
A schematic diagram of equipment setup.

The GP3 eye-tracker hardware unit was placed about 65 cm from the eye of test subjects. In all cases, I tried to ensure that the illumination of the test subjects was naturally uniform and that no sudden changes in light conditions occurred. I displayed the algorithm descriptions and the source code editing interface of the Visual Studio development environment on a 22″ diameter LG22M45 monitor capable of 1920 × 1080. A schematic diagram of the testing environment is shown in Figure 2.

**Figure 3:**
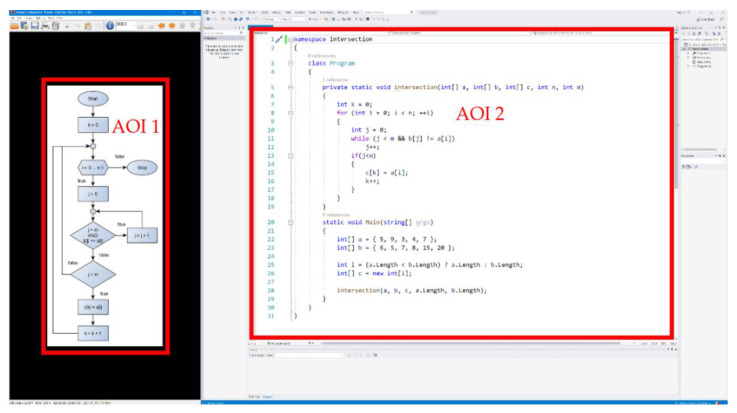
Definition of AOIs.

To obtain additional information on eye movement parameters, I marked these areas AOI in OGAMA, as illustrated in Figure 3. On the left side (AOI1) of the display is an image of the algorithm to be implemented represented by a randomly selected algorithm description tool (FCh: AOI1_FCh_ or N-S diagram: AOI1_N-S_), while on the right side (AOI2) it was possible to implement the source code (FCh-based implementation: AOI2_FCh-based implementation_ and N-S-based implementation: AOI2_N-S-based implementation_). The fixation detection algorithms of OGAMA come from LC Technologies, which is a dispersion-type algorithm with window and was ported to C# and a time estimation support was added. A detailed description of the algorithm can be found in reference [34]. 

The algorithm description tools randomly represented one of the algorithms detailed in Section 3.3. In any case, in order to avoid that level of the test subject’s knowledge concerning the results of the research, each test subject must also implement an algorithm on 1 FCh and 1 N-S. The density of the figures or texts also affects the processing of information [35], so clarity and easy readability are important factors in the algorithm description tools and program codes; therefore, I tried to keep the same distance between the texts. A total of 42 FChs and 42 N-S-based eye movement parameter packages were saved in a database for further evaluation.

**Figure 4:**
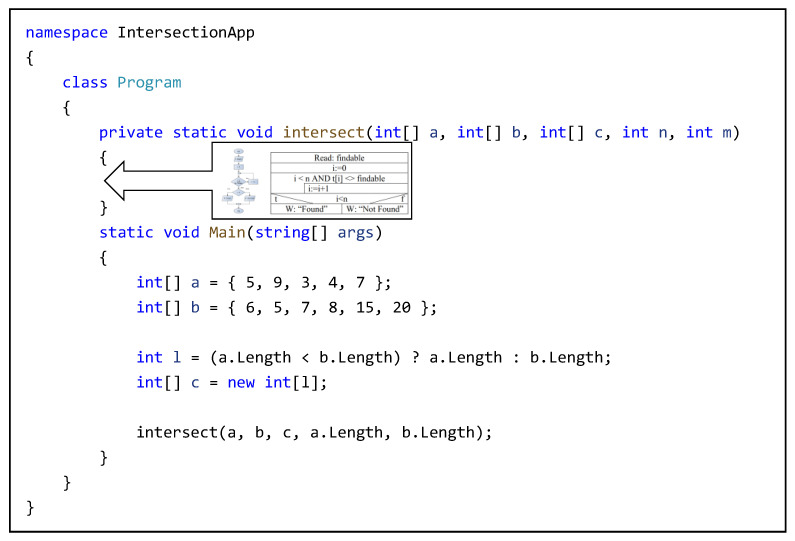
The available source code snippet.

During the implementation, all that was needed was to implement the algorithm, as the additional source code snippet was already available to everyone. Figure 4 illustrates such a snippet of code.

### 3.3. Applied Algorithms

Test subjects had to implement randomly selected algorithms already learned in their previous studies in C# based on an FCh or N-S. During the implementation of the Decision algorithm, it had to be decided whether an element (findable) with a given property could be found in the array (*T*) incoming in the parameter. As soon as the test condition was met, the applicable cycle had to stop as it would have been unnecessary to continue running. If the loop had stopped because we also exceeded the last test element in the array, the element we were looking for could not be found in the array. The pseudo-description of the algorithm is shown in Algorithm 1.
**Algorithm 1: Possible Implementation of Decision**1: **data:** *T*: input array; *N*: length of array; *findable*: value to look for. 2: **procedure** Decision (*T*, *N*, *findable*) 3:  **while** *i* ≤ *N and T*[*i*] ≠ *findable* **do** 4:   *i* ← *i* + 1 5:  **end while** 6:  **if** *i* ≤ *N* **then** 7:   **output:** “Found” 8:  **else** 9:   **output:** “Not Found” 10:  **end if** 11: **end procedure**

During the implementation of the Intersection algorithm, the identical elements of two arrays (*A*[1 … *N*] és *B*[1 ... *M*]) had to be selected into a third, C array. The problem to be solved can only be interpreted accurately if an element is not found twice in each array. The maximum number of elements in array C is the smaller of *N* and *M*. The pseudo-description of the algorithm is shown in Algorithm 2.
**Algorithm 2: Possible Implementation of Intersection**1: **data:** *A*, *B*, *C*: input arrays; *N*: length of *A*; *M*: length of *B* 2: **procedure** Intersection (*A*, *B*, *C*, *N*, *M*) 3:  *k* ← 0 4:  **for** *i* ← 1 to *N* **do** 5:   *j* ← 0 6:   **while** *j* ≤ *M and B*[*j*] ≠ *A*[*i*] **do** 7:    *j* ← *j* + 1 8:   **end while** 9:   **if** *j* ≤ *M* **then** 10:    *c*[*k*] ← *a*[*i*] 11:    *k* ← *k* + 1 12:   **end if** 13:  **end for** 14: **end procedure**

During the implementation of the Union algorithm, the elements of two arrays (*A*[1 … *N*] and *B*[1 ... *M*]) that are in at least one of the arrays are placed in a third array C. The pseudo-description of the algorithm is shown in Algorithm 3.
**Algorithm 3: Possible Implementation of Union**1: **data:** *A*, *B*, *C*: input arrays; *N*: length of *A*; *M*: length of *B* 2: **procedure** Union (*A*, *B*, *C*, *N*, *M*) 3:  **for** *i* ← 1 to *N* **do** 4:   *c*[*i*] ← *a*[*i*] 5:  *k* ← *n* 6:  **for** *j* ← 1 to *M* **do** 7:   *i* ← 0 8:   **while** *i* ≤ *N and B*[*i*] ≠ *A*[*i*] **do** 9:    *i* ← *i* + 1 10:   **end while** 11:   **if** *i* ≥ *N* **then** 12:    *c*[*k*] ← *b*[*j*] 13:    *k* ← *k* + 1 14:   **end if** 15:  **end for** 16: **end procedure**

## 4. Results

The results were determined from the eye movement parameters observed and recorded in all AOI areas. In selecting the appropriate statistical tests, it was considered that the test subjects were independent of each other and the same subjects within a group were examined. It was also determined that, for the examination of the distribution of the variables, the Shapiro–Wilk test was be applied. In case of the applied statistical tests, *p* < 0.05 value was determined as significant.

### 4.1. Fixation Duration Mean Based on AOIs

A brief descriptive statistic of the fixation duration mean results in both AOIs is summarized in Table 1 and Table 2.

**Figure 5:**
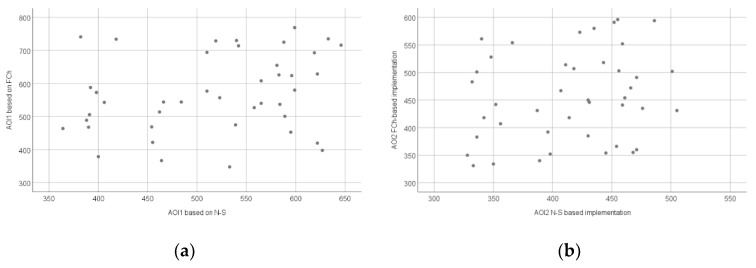
Scatter plot of the fixation duration mean (milliseconds) based on all AOIs: AOI1 (**a**) and AOI2 (**b**).

Figure 5a shows the relationship between the fixation duration mean measured during the interpretation of the FCh and N-S diagrams in the AOI1 area, while Figure 5b shows the relationship between the fixation duration mean measured during the implementation of the source codes based on the FCh and N-S diagrams in the AOI2 area.

As a further part of the evaluation, a statistical test was used to determine whether the recorded data differed significantly.

The normality results of the fixation duration mean measured in the AOI1 area are significant (AOI1_FCh_: *W*(42) = 0.957, *p* = 0.111, AOI1_N-S_: *W*(42) = 0.920, *p* = 0.006); therefore the Wilcoxon signed-rank test was applied (AOI1: *T* = −242, *Z* = 2.62, *p* = 0.009 (2-tailed), *r* = 0.286), based on which it can be stated that the fixation duration mean was significantly longer with a small effect in the case in which AOI1_fc_: *Mdn* = 550.5 milliseconds than in case in which AOI1_N-S_: *Mdn* = 536 milliseconds.

In AOI2 the normality results are significant too (AOI2_FCh-based implementation_: *W*(42) = 0.952, *p* = 0.078, AOI2_N-S-based implementation_: *W*(42) = 0.928, *p* = 0.011), so the Wilcoxon signed-rank test was used as well (AOI2: *T* = −231, *Z* = 2.75, *p* = 0.006 (2-tailed), *r* = 0.3), based on which it can be stated that the fixation duration mean was significantly longer with a medium effect in the case in which AOI2_FCh-based implementation_: *Mdn* = 448 milliseconds than in the case in which AOI2_N-S-based implementation_: *M* = 426.5 milliseconds.

**Figure 6:**
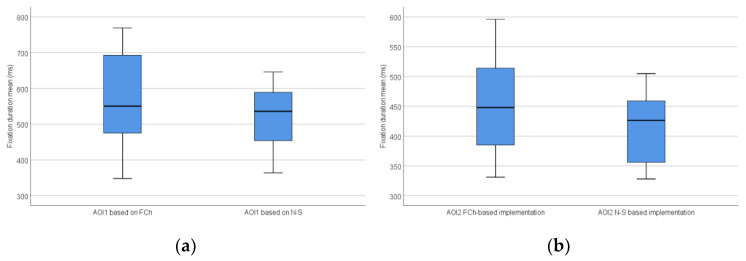
The distribution of the fixation duration mean (milliseconds) based on all AOIs: AOI1; (**a**) and AOI2; (**b**).

The distribution of the fixation duration mean based on all AOIs is shown in Figure 6.

In response to the RQ1 question, there was a significant difference in the fixation duration mean (*p* = 0.009) when the test subjects examined and studied different types of algorithm descriptions to be implemented. The results show that in the case of the N-S, the algorithm to be implemented can be interpreted with a shorter fixation result, therefore a lower cognition load, as opposed to the FCh-based description.

In response to the RQ2 question, there was a significant difference in the fixation duration mean (*p* = 0.006) when the test subjects implemented the algorithms to be implemented based on different types of algorithm descriptions, The results show that using the N-S diagram-based algorithm description, the implementation can be performed with a shorter fixation duration mean, so lower cognition load, as opposed to the FCh-based implementation.

### 4.2. Number of Fixations Based on AOIs

A brief descriptive statistic of the number of fixations results in both AOIs is summarized in Table 3 and Table 4.

**Figure 7:**
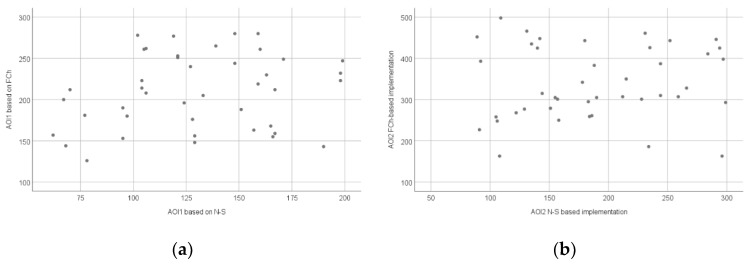
Scatter plot of the number of fixations (count) based on all AOIs: AOI1 (**a**) and AOI2 (**b**).

Figure 7a shows the relationship between the number of fixations measured during the interpretation of the FCh and N-S diagrams in the AOI1 area, while Figure 7b shows the relationship between the number of fixations measured during the implementation of the source codes based on the FCh and N-S diagrams in the AOI2 area.

As a further part of the evaluation, a statistical test was used to determine whether the recorded data differed significantly. The normality results of the number of fixations measured in the AOI1 area are not significant (AOI1_FCh_: *W*(42) = 0.953, *p* = 0.081, AOI1_N-S_: *W*(42) = 0.965, *p* = 0.230), so a paired sample *t*-test was used (AOI1: *t*(41) = 9.786, *p* < 0.001 (2-tailed), *r* = 0.837), based on which it can be stated that the number of fixations was significantly more with a large effect in the case in which AOI1_FCh_: *M* = 209.74, *SD* = 44.74 than in the case in which AOI1_N-S_: *M* = 130.29, *SD* = 38.08.

In AOI2 the normality results are significant (AOI2_FCh-based implementation_: *W*(42) = 0.952, *p* = 0.077, AOI2_N-S-based implementation_: *W*(42) = 0.942, *p* = 0.034), so the Wilcoxon signed-rank test was applied (AOI2: *T* = 21.5, *Z* = 5.377, *p* < 0.001 (2-tailed), *r* = 0.587), based on which it can be stated that the number of fixations was significantly more with a large effect in the case in which AOI2_FCh-based implementation_: *Mdn* = 312.5 than in the case in which AOI2_N-S-based implementation_: *Mdn* = 183.5.

**Figure 8:**
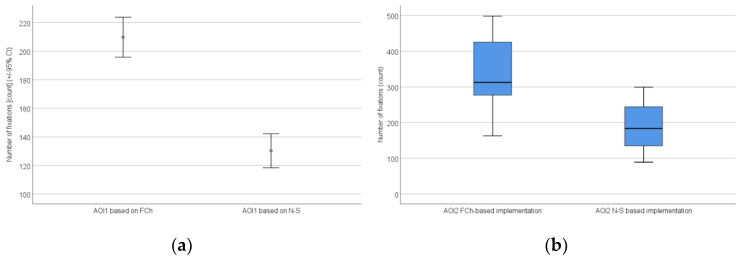
The confidence intervals (**a**) and the distribution (**b**) of the number of fixations based on all AOIs: AOI1 (**a**) and AOI2 (**b**).

The confidence intervals and the distribution of the number of fixations based on all AOIs are shown in Figure 8.

In response to RQ1, there was a significant difference in the number of fixations (*p* < 0.001) when the test subjects examined and studied the different types of algorithm descriptions to be implemented. The results show that in the case of the N-S, the algorithm to be implemented can be interpreted with less fixations, therefore a lower cognition load, as opposed to the FCh-based description.

In response to RQ2, there was a significant difference in the number of fixations (*p* < 0.001) when the test subjects implemented the algorithms to be implemented based on different types of algorithm descriptions. The results show that, using the N-S-based algorithm description, the implementation can be performed with less fixations, so a lower cognition load, compared to the FCh-based implementation.

### 4.3. Average of Pupil Diameter Based on AOIs

A brief descriptive statistic of the average of pupil diameter results in both AOIs is summarized in Table 5 and Table 6.

**Figure 9:**
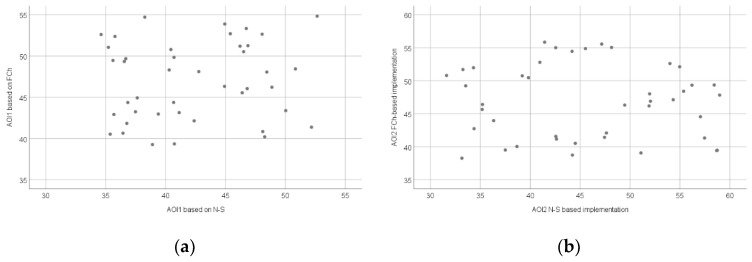
Scatter plot of the average of pupil diameter based on all AOIs: AOI1 (**a**) and AOI2 (**b**).

Figure 9a shows the relationship between the average of pupil diameter measured during the interpretation of the FCh and N-S diagrams in the AOI1 area, while Figure 9b shows the relationship between the average of pupil diameter measured during the implementation of the source codes based on the FCh and N-S diagrams in the AOI2 area.

As a further part of the evaluation, a statistical test was used to determine whether the recorded data differed significantly. The normality results of the average of pupil diameter measured in the AOI1 area are significant (AOI1_FCh_: *W*(42) = 0.947, *p* = 0.050, AOI1_N-S_: *W*(42) = 0.928, *p* = 0.011); therefore the Wilcoxon signed-rank test was applied (AOI1: *T* = −156, *Z* = 3.695, *p* < 0.001 (2-tailed), *r* = 0.403), based on which it can be stated that the average of pupil diameter was significantly larger with a medium effect in the case in which AOI1_FCh_: *Mdn* = 47.195 pixels than in the case in which AOI1_N-S_: *Mdn* = 40.930 pixels.

In AOI2 the normality results are significant (AOI2_fc based implementation_: *W*(42) = 0.938, *p* = 0.025, AOI2_N-S-based implementation_: *W*(42) = 0.940, *p* = 0.028), so the Wilcoxon signed-rank test was used (AOI2: *T* = −391.5, *Z* = 0.750, *p* = 0.453 (2-tailed), *r* = 0.082), based on which it can be stated that the average of pupil diameter was not significantly larger in the case in which AOI2_FCh-based implementation_: *Mdn* = 47.025 pixels than in the case in which AOI2_N-S-based implementation_: *Mdn* = 45.020 pixels.

**Figure 10:**
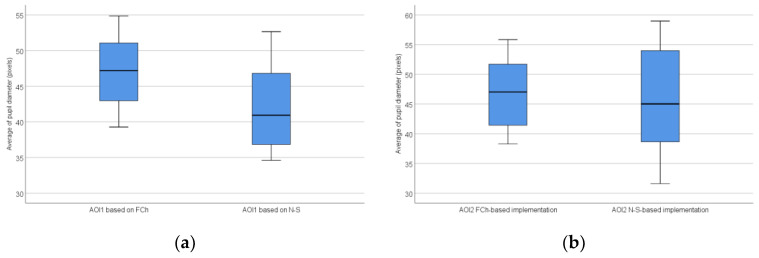
The distribution of the average of pupil diameter based on all AOIs: AOI1 (**a**) and AOI2 (**b**).

The distribution of the average of pupil diameter based on all AOIs is shown in Figure 10.

In response to RQ1, the average of pupil diameter showed a significant difference (*p* < 0.001) when the test subjects studied the different types of algorithm descriptions to be implemented. The results show that in the case of N-S, the average of pupil diameter is smaller, so the algorithm to be implemented can be interpreted with a lower cognition load, as opposed to the FCh-based description.

In response to RQ2, in the average of pupil diameter there was not a significant difference (*p* = 0.453) when the test subjects used an N-S or FCh in the implementation of the algorithm to be implemented, so using this eye-tracking parameter it cannot be clearly stated that the implementation can be performed with a different cognition load; however, a kind of tendency can be seen here as well, during which it can be seen that by interpreting the N-S, the implementation can be implemented, if not significantly, with a slightly lower mental load than using the FCh.

## 5. Discussion

After a detailed evaluation of the results of the eye tracking parameters recorded, it can be stated that the N-S can be read and interpreted with less cognitive load, and the implementation of the algorithms can be done with a lower mental load. In the research, the number of fixations, fixation duration means and pupil diameters, which indicate the level of attention and cognitive condition of the test subjects, also suggest this. This is because in the case of N-S, significantly less and shorter information recording and processing were necessary to understand and implement the algorithm.

In addition, after testing, in a short interview, test subjects summarized their experiences and shared what they thought about which algorithm descriptive tool best supported their work. The responses received and the opinions expressed clearly support what was revealed and described in the evaluation of the results. It was said that the length of the FCh and the diversity of the arrows made it difficult to understand how the algorithm works. Using the more concise N-S, for example, they were able to decide much more clearly and in less time how long a loop would last.

The results revealed and defined in the research can greatly contribute to the more efficient completion of the implementation phase of the software development lifecycle at a lower cost. This may be especially true for programming learners and beginner programmers. When developing an application, the right tools can help you teach and learn more efficiently and effectively. It can be stated that in order to increase the efficiency of knowledge transfer and learning in the field of education, it is worth introducing the N-S from the beginning of programming teaching, thus making the understanding of an algorithm more efficient with lower cognitive load, avoiding difficulties and problems occurring from the understanding and readability of the syntax. In addition to reducing the costs of each development phase in the industry, we can create a source code that is easier to read and maintain if we always choose the right tool or programming technique. Devices that record the route of such a gaze can help in this. Apparently, testing may seem like a waste of time at the beginning of the software development phase, but it can pay off later, even in the implementation phase, and further phases such as testing and evolution can be significantly decreased.

In addition, the reliability and accuracy of hardware and software devices that record eye movement parameters are improving, and their costs are decreasing. They can facilitate the selection of personalized or team-specific support tools, programming techniques and technologies based on recorded and evaluated eye movement results. This improves the collaboration and communication of development teams and further optimizes their coding styles. Eventually, the evolution of software, its upgradeability and its maintainability can be significantly improved, and their costs can be decreased.

In the future, with the applied eye movement tracking system, additional programming tools, techniques and technologies may become available for cognitive load. For students in education, and for industry development teams in the industry, the use of tools, methods, techniques and technologies that require less mental effort can result in a more effective learning and development process. In further research, using the developed eye movement tracking system, I would like to investigate additional programming tools, methods, solutions, techniques and technologies in terms of mental load in relation to eye movement parameters.

## 6. Conclusions

Research based on eye movement tracking is expected to emerge in the near future for the study of cognitive processes such as programming. The study examined the readability and interpretability of two algorithm description tools: The FCh and the N-S involving test subjects. In the study, an eye-tracking-based test system was developed, with the help of which the gaze path of the test subjects can be observed, recorded and evaluated, and then conclusions can be drawn after determining the obtained results. The article explores the relationships between the cognitive load and eye movement parameters of test subjects and the results revealed using the test system, and emphasizes the importance and significance of properly selected tools, programming techniques and technologies.

## Figures and Tables

**Table 1:** The descriptive statistic of the fixation duration mean based on AOI1 (*N* = 42).

AOI1_FCh_ (Milliseconds)				AOI1_N-S_ (Milliseconds)			
Min	Max	Mean	SD	Min	Max	Mean	SD
348	769	569.2	117.8	364	646	516.69	86.27

**Table 2:** The descriptive statistic of the fixation duration mean based on AOI2 (*N* = 42).

AOI2_FCh-based implementation_ (Milliseconds)				AOI2_N-S-based implementation_ (Milliseconds)			
Min	Max	Mean	SD	Min	Max	Mean	SD
331	596	456.24	80.46	328	505	414.67	53.27

**Table 3:** The descriptive statistic of the number of fixations based on AOI1 (*N* = 42).

AOI1_FCh_ (Count)				AOI1_N-S_ (Count)			
Min	Max	Mean	SD	Min	Max	Mean	SD
126	280	209.7	44.74	62	199	130.29	38.08

**Table 4:** The descriptive statistic of the number of fixations based on AOI2 (*N* = 42).

AOI2_FCh-based implementation_ (Count)				AOI2_N-S-based implementation_ (Count)			
Min	Max	Mean	SD	Min	Max	Mean	SD
163	498	339	88.35	89	299	188.9	65.69

**Table 5:** The descriptive statistic of the average of pupil diameter in AOI1 (*N* = 42).

AOI1_FCh_ (Pixels)				AOI1_N-S_ (Pixels)			
Min	Max	Mean	SD	Min	Max	Mean	SD
39.28	54.84	46.98	4.71	34.61	52.65	42.35	5.41

**Table 6:** The descriptive statistic of the average of pupil diameter in AOI2 (*N* = 42).

AOI2_FCh-based implementation_ (Pixels)				AOI2_N-S-based implementation_ (Pixels)			
Min	Max	Mean	SD	Min	Max	Mean	SD
38.28	55.85	46.88	5.52	31.61	58.97	42.75	8.62

## Data Availability

Not applicable.
